# Supplementary material for: Long-term statins administration exacerbates diabetic nephropathy via ectopic fat deposition in diabetic mice
Source: Nat Commun. 2023 Jan 24;14:390. doi: 10.1038/s41467-023-35944-z (PMC9873739; doi:10.1038/s41467-023-35944-z)
Supplement: Supplementary file 1 — Supplementary Information [file 41467_2023_35944_MOESM1_ESM.pdf]

Long-term statins administration exacerbates diabetic nephropathy via ectopic fat deposition in diabetic mice

Tong-sheng Huang[1,2 #], Teng Wu[1,2 #], Yan-di Wu[1,2], Xing-hui Li[1,2], Jing Tan[1,2], Cong-hui Shen[1,2], Shi-jie Xiong[1,2], Zi-qi Feng[1,2], Sai-fei Gao[1], Hui Li[1], Wei-bin Cai[1,2,3 *]

[1] Guangdong Engineering & Technology Research Center for Disease-Model Animals, Laboratory Animal Center, Zhongshan School of Medicine, Sun Yat-sen University, Guangzhou 510080, Guangdong, China.

[2] Department of Biochemistry, Zhongshan School of Medicine, Sun Yat-sen University, Guangzhou 510080, Guangdong, China.

[3] Guangdong Provincial Key Laboratory of Digestive Cancer Research, The Seventh Affiliated Hospital of Sun Yat-sen University, Shenzhen, 518107, Guangdong, China.

These authors contributed equally: Tong-sheng Huang, Teng Wu.

*Correspondence to: Professor. Wei-bin Cai, Sun Yat-sen University, Guangzhou 510080, PR China (email: caiwb@mail.sysu.edu.cn).

Supplementary Table 1: CK-MB and Myoglobin of *db/db* mice treated with statin. All mice were approximately 50 weeks old. *n* = 6 or 9 in each group. One-way ANOVA with Tukey post hoc test was used for the analysis of statistical significance. Data are expressed as means ± SEM. As for S-CK-MB, compared with Db/m, *P* = 0.002 (Db), *P* = 0.007 (Db+Ato5), *P* = 0.005 (Db+Ato10), *P* = 0.006 (Db+Rosu20). Significance **P* < 0.05 versus Db/m group; ***P* < 0.01 versus Db/m group. Source data are provided as a Source Data file.

Groups	Db/m	Db	Db+Ato5	Db+Ato10	Db+Rosu20
S-CK-MB (ng/mL)	15.32±8.10	215.82±112.25**	210.05±106.94**	220.35±132.20**	226.31±147.40**
S-Myoglobin(ng/mL)	36.01±2.70	37.97±3.82	36.76±4.30	38.07±2.90	37.99±2.59

Supplementary Table 2: Leptin and Insulin of *db/db* mice treated with statin. All mice were approximately 50 weeks old. *n* = 6 or 9 in each group. One-way ANOVA with Tukey post hoc test was used for the analysis of statistical significance. Data are expressed as means ±SEM. As for S-Leptin, compared with Db/m, *P* < 0.001 (Db), *P* < 0.001 (Db+Ato5), *P* < 0.001 (Db+Ato10), *P* < 0.001 (Db+Rosu20); compared with Db, *P* < 0.001 (Db+Ato5), *P* = 0.001 (Db+Ato10), *P* = 0.001 (Db+Rosu20). As for S-Insulin, compared with Db/m, *P* < 0.001 (Db), *P* < 0.001 (Db+Ato5), *P* < 0.001 (Db+Ato10), *P* < 0.001 (Db+Rosu20). Significance **P* < 0.05 versus Db/m group; *****P* < 0.0001 versus Db/m group. #*P* < 0.05 versus Db group; ##*P* < 0.01 versus Db group; ###*P* < 0.001 versus Db group, ####*P* < 0.0001 versus Db group. Source data are provided as a Source Data file.

Groups	Db/m	Db	Db+Ato5	Db+Ato10	Db+Rosu20
S-Leptin(ng/mL)	3.90±0.827	40.57±6.89****	37.36±21.68****	39.80±10.36****	42.20±24.24****
S-Insulin (mIU/mL)	7.25±1.44	28.93±6.41****	49.90±9.20****	44.90±8.82****	46.83±9.80****

Supplementary Table 3: Biochemical profiles of STZ/HFD-induced diabetic mice treated with statin. All mice were approximately 50 weeks old. *n* = 6 in each group. One-way ANOVA with Tukey post hoc test was used for the analysis of statistical significance. Data are expressed as means ±SEM. As for S-Leptin, compared with Db/m, $P = 0.002$ (Db), $P < 0.001$ (Db+Ato5), $P < 0.001$ (Db+Ato10), $P = 0.002$ (Db+Rosu20); compared with Db, $P = 0.014$ (Db+Ato10). As for S-Adiponectin, compared with Db/m, $P = 0.004$ (Db), $P = 0.007$ (Db+Ato5), $P = 0.002$ (Db+Ato10), $P = 0.001$ (Db+Rosu20). As for S-Chol, compared with Db/m, $P < 0.001$ (Db), $P = 0.170$ (Db+Ato5), $P = 0.023$ (Db+Ato10), $P = 0.072$ (Db+Rosu20); compared with Db, $P = 0.004$ (Db+Ato5), $P = 0.012$ (Db+Ato10), $P = 0.012$ (Db+Rosu20). As for S-TG, compared with Db/m, $P < 0.001$ (Db), $P = 0.098$ (Db+Ato5); compared with Db, $P = 0.003$ (Db+Ato5), $P < 0.001$ (Db+Ato10), $P < 0.001$(Db+Rosu20). As for S-LDL-C, compared with Db/m, $P = 0.048$ (Db). As for S-Cr, compared with Db/m, $P = 0.160$ (Db), $P = 0.023$ (Db+Ato5), $P = 0.002$ (Db+Ato10), $P < 0.001$ (Db+Rosu20); compared with Db, $P = 0.009$ (Db+Ato10), $P = 0.001$ (Db+Rosu20). As for S-AST, compared with Db/m, $P < 0.001$ (Db), $P < 0.001$ (Db+Ato5), $P = 0.001$ (Db+Ato10), $P = 0.001$ (Db+Rosu20); compared with Db, $P = 0.028$ (Db+Ato10), $P = 0.059$ (Db+Rosu20). As for S-ALT, compared with

Db/m, *P* < 0.001 (Db), *P* = 0.003 (Db+Ato5), *P* < 0.001 (Db+Ato10), *P* = 0.001 (Db+Rosu20); compared with Db, *P* = 0.029 (Db+Ato5), *P* = 0.095 (Db+Ato10), *P* = 0.103 (Db+Rosu20). Significance **P* < 0.05 versus Db/m group; ***P* < 0.01 versus Db/m group; ****P* < 0.001 versus Db/m group, *****P* < 0.0001 versus Db/m group. #*P* < 0.05 versus Db group; ##*P* < 0.01 versus Db group; ###*P* < 0.001 versus Db group, ####*P* < 0.0001 versus Db group. Source data are provided as a Source Data file.

Groups	Con	Stz	Stz+Ato5	Stz+Ato10	Stz+Rosu20
S-Insulin（mIU/mL）	7.26±1.44	37.36±8.16****	40.54±11.51****	51.92±10.91****#	37.24±16.29****
S-Adiponectin (pg/mL)	36.92±4.30	31.37±3.07***	31.76±2.82***	31.23±3.59***	30.36±4.57***
S-Leptin(ng/mL)	3.80±0.81	3.39±0.58	3.53±0.57	3.43±0.45	4.17±0.47##
S-Chol (mmol/L)	1.93±0.47	4.24±1.36****	2.61±0.81##	2.96±0.90*#	2.83±0.73#
S-TG (mmol/L)	0.52±0.07	0.87±0.23****	0.64±0.13##	0.54±0.08####	0.57±0.05####
S-LDL-C (mmol/L)	0.46±0.09	0.73±0.24*	0.64±0.39	0.51±0.20	0.56±0.11
S-HDL (mg/dL)	2.65±0.33	1.70±0.57	2.48±1.10	2.36±0.70	2.47±0.91
S-Cr (μmol/L)	29.88±4.16	48.06±12.26****	55.97±9.84*	75.17±27.93****##	68.82±8.36***#
S-AST(U/L)	11.56±7.29	36.45±9.58****	30.77±5.37****	26.21±8.35****#	27.22±7.65****
S-ALT (U/L)	11.16±4.21	35.97±7.29***	25.10±6.99**#	28.42±11.37***#	27.99±4.85***#

Supplementary Table 4: Antibody Information.

Antibodies	Manufacture and item NO.	Application
α-SMA	Cell signaling technology(19245S)	IHC
COL1A1	Cell signaling technology(72026S)	IHC
NF-κB	Cell signaling technology(8242S)	IF
CD68	Cell signaling technology(97778S)	IHC
4-HNE	Abcam(ab46545)	IHC
IL-1β	Cell signaling technology(12242S)	WB, IHC
NGAL	Abcam (ab125075)	WB, IHC
SREBP-1	Novus Biologicals (NB100-2215)	WB, IHC
Akt (pan) (C67E7)	Cell signaling technology (4691S)	WB
Phospho-Akt (Ser473)	Cell signaling technology (4060S)	WB
Phospho-Akt (Thr308) (244F9)	Cell signaling technology (4056S)	WB
FAS	Cell signaling technology (3180S)	WB, IHC
SCD1	Cell signaling technology (2794S)	WB, IHC
ACC1	Proteintech (21923-1-AP)	WB
Phospho-p70 S6 Kinase (Thr389)	Cell signaling technology (9205S)	WB
p70(S6K) Polyclonal antibody	Proteintech (14485-1-AP)	WB
PI3 Kinase p85 (19H8)	Cell signaling technology (4257S)	WB
Phospho-PI3 Kinase p85 (Tyr458)/p55 (Tyr199)	Cell signaling technology (4228S)	WB
ATGL (30A4)	Cell signaling technology (2439S)	WB
CD36	Sigma aldrich (HPA002018)	WB, IHC
CPT1α	Cell signaling technology (12252S)	WB
RAGE	Proteintech (16346-1-AP)	IHC
LDLR	Proteintech (10785-1-AP)	IHC, WB
Anti-Wilms Tumor	Abcam (ab89901)	IF
Nephrin	R&D (AF3159)	IF
PPARγ (81B8)	Cell signaling technology (2443S)	WB
HMGCR	Abcam (ab242315)	IHC, WB

Supplementary Table 5: IHC-related Antibody Information

Antibody	Labeling position	Subcellular localization	IHC in Figure
α-SMA	Myofibroblasts	Cytoplasm, Cytoskeleton	Fig 3d
COL1A1	ECM	Extracellular matrix	Fig 3c
NF-κB	Podocytes and tubular epithelial cell	Nucleus, Cytoplasm.	Fig 4b
CD68	Macrophages	Cell membrane, Endosome, Lysosome, Membrane	Fig 4a
4-HNE	Podocytes and tubular epithelial cell	Cytoplasmic	Fig 5f & Supplemental Figure 7d
IL-1β	Podocytes and tubular epithelial cell, ECM	Cytoplasm, Lysosome, Secreted	Fig 4d
NGAL	Tubular epithelial cell, ECM	Cytoplasmic, Secreted	Fig 4c
SREBP-1	Podocytes and tubular epithelial cell	Membranes of ER, Golgi and Cytoplasmic Vesicles. Processed SREBP1 (1-490 a.a.), translocate to the nucleus.	Fig 7a
FAS	Podocytes and tubular epithelial cell	Endoplasmic reticulum, Membrane	Fig 7b
SCD1	Podocytes and tubular epithelial cell	Endoplasmic reticulum membrane.	Fig 7c
LDLR	Podocytes and tubular epithelial cell	Cell membrane, Coated pit, Endosome, Golgi apparatus, LDL, Lysosome, Membrane	Fig 6c & Supplemental Figure 2d
Anti-Wilms Tumor	Podocytes	Nucleus.	Fig 2e
Nephrin	Podocytes	Cell membrane, Membrane	Fig 2f
CD36	Podocytes and tubular epithelial cell	Cell membrane. Membrane raft. Golgi apparatus. Apical cell membrane.	Fig 6d
RAGE	Podocytes and tubular epithelial cell, ECM	Secreted and Cell membrane.	Fig 1g & Supplemental Figure 7c
HMGCR	Podocytes and tubular epithelial cell	Endoplasmic reticulum membrane. Peroxisome membrane.	Fig 6b & Supplemental Figure 2c

Supplementary figure

a.

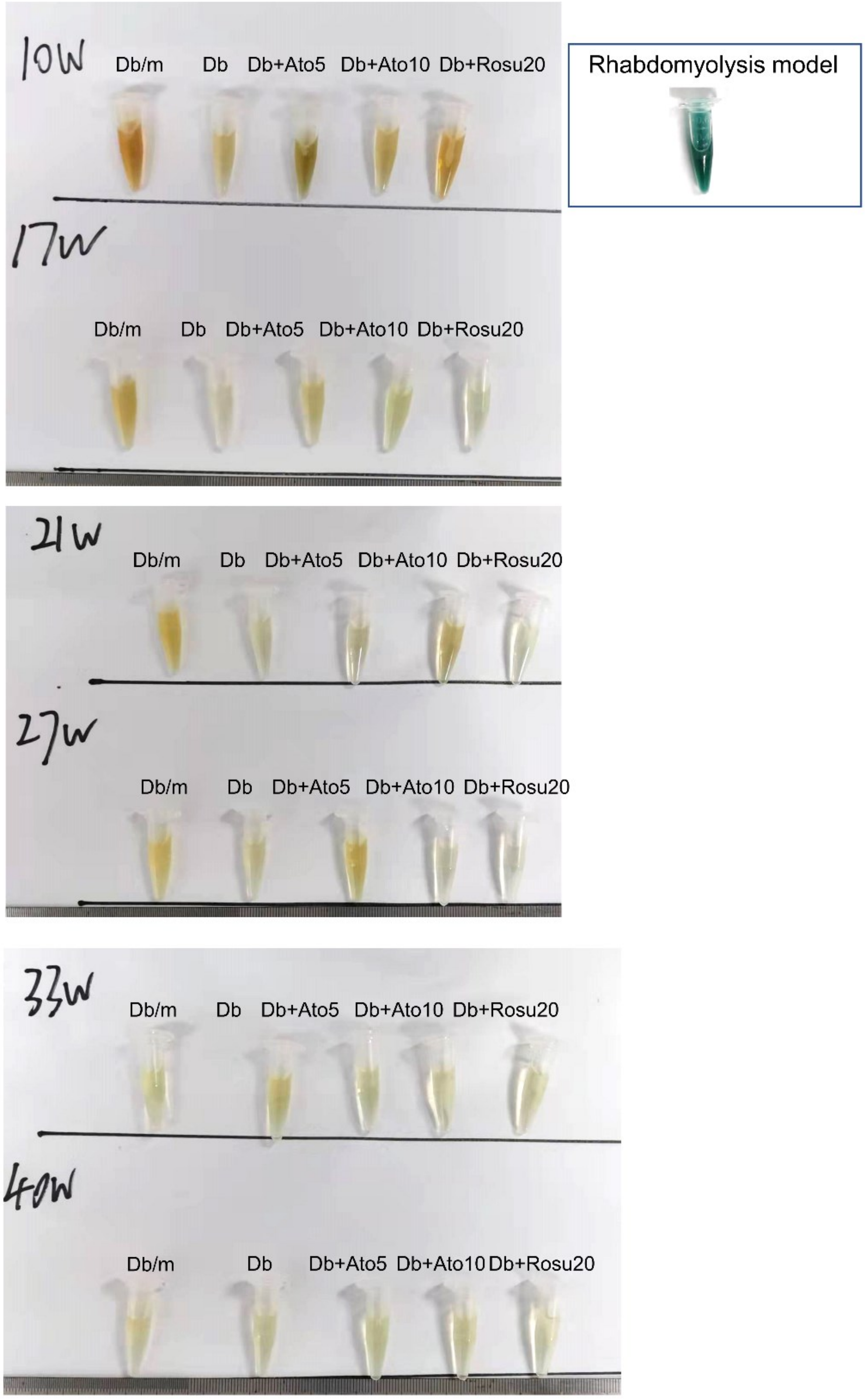

Supplementary Figure 1. Long-term statins administration did not cause hemoglobinuria due to myolysis. (a) Time-course of urinary hemoglobin qualitative detection. Source data are provided as a Source Data file.

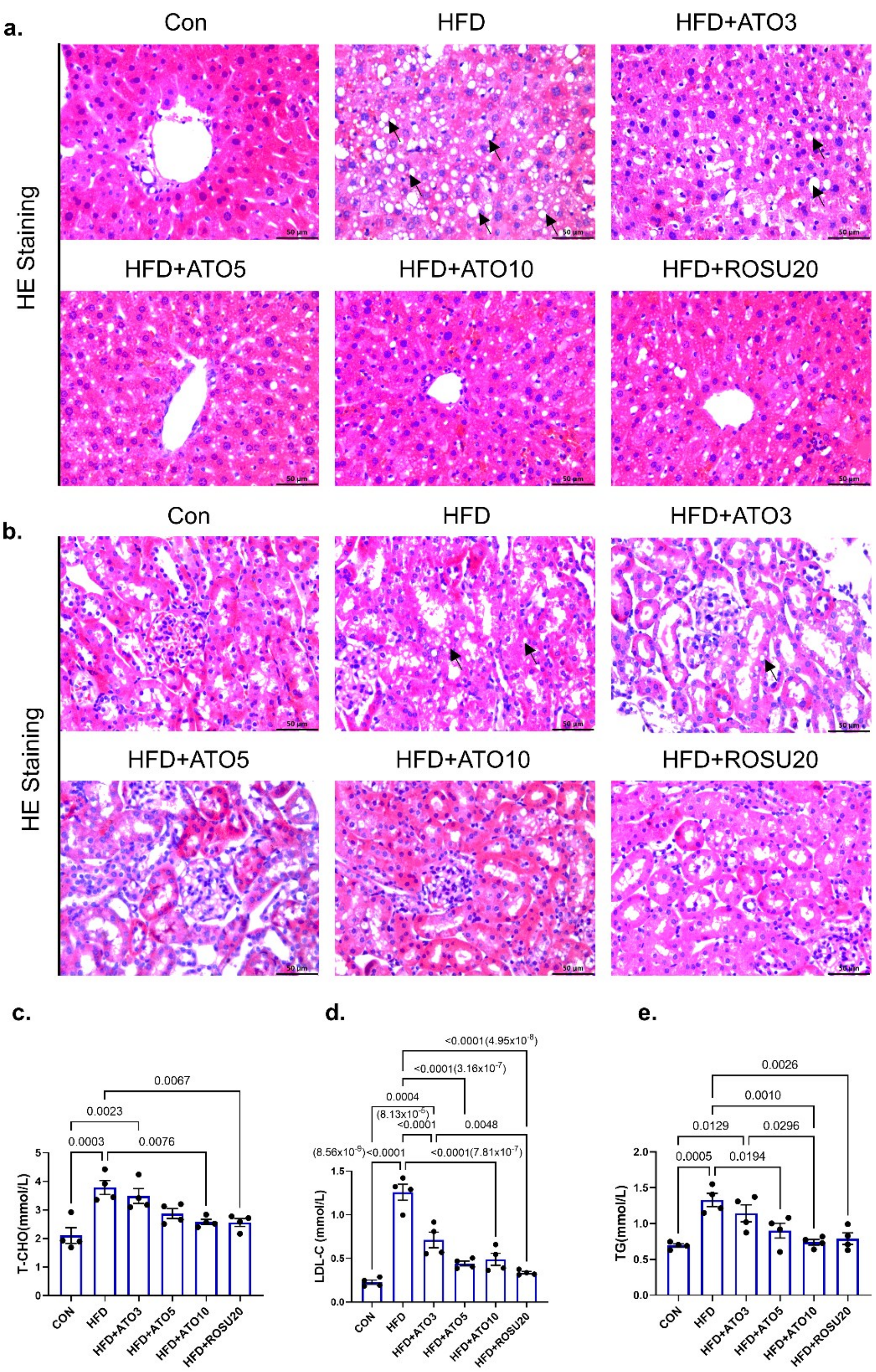

Supplementary Figure 2. Pathological characteristics and lipid profile of long-term statins administration in HFD mice. All mice were approximately 50 weeks old. **(a)** Representative image of HE staining for liver. Arrows point to areas of hepatocyte steatosis. Original magnification ×400. Scale bar: 50 μm. **(b)** Representative image of HE staining for kidney. Arrows indicate

degeneration and vacuolization of renal tubular epithelial cells. Original magnification ×400. Scale bar: 50 μm. **(c-e)** Lipid profile in *db/db* mice. All image part of the kidney was cortex. Data are expressed as means ± SEM. *n* = 4 in each group. One-way ANOVA with Tukey post hoc test was used for the analysis of statistical significance. Source data are provided as a Source Data file.

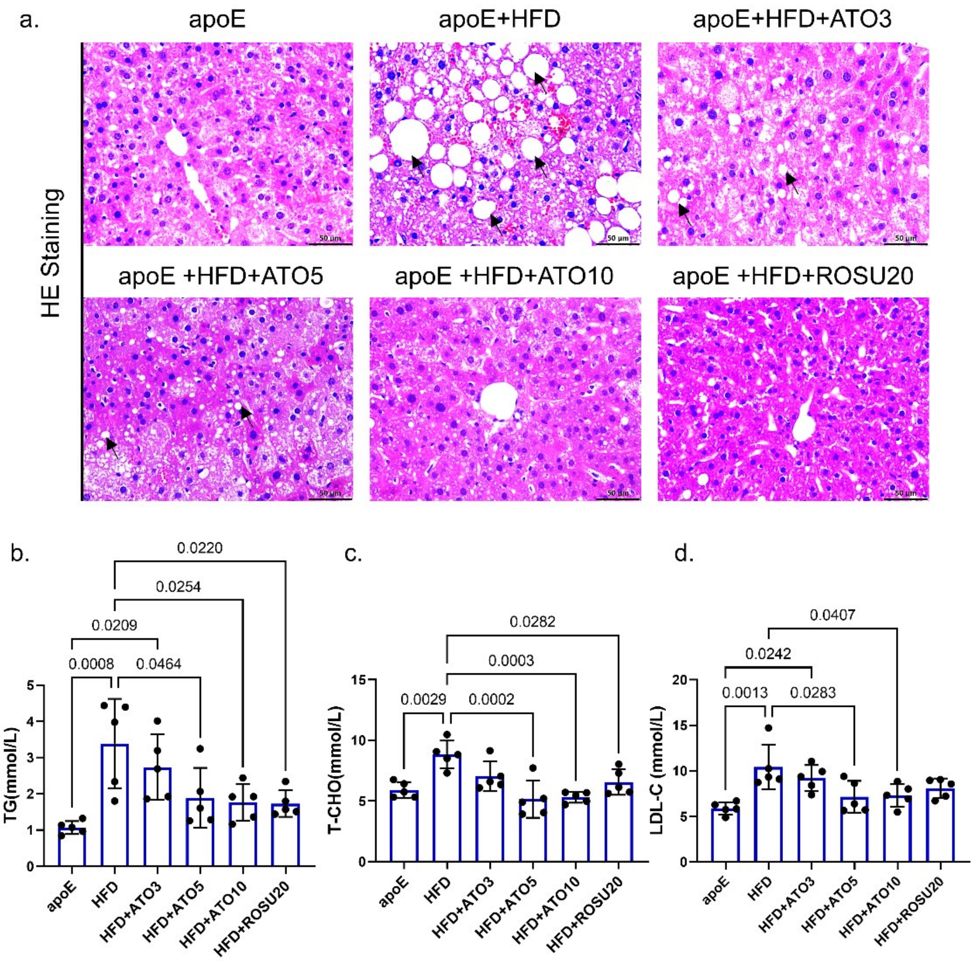

Supplementary Figure 3. Pathological characteristics and lipid profile of long-term administration of statins in HFD *ApoE*$^{-/-}$ mice. (a) Representative image of HE staining for liver. Arrows indicate hepatocyte steatosis. Original

magnification ×400. Scale bar: 50 μm. **(b-d)** Lipid profile in *db/db* mice. Data are expressed as means ± SEM. n = 5 in each group. One-way ANOVA with Tukey post hoc test was used for the analysis of statistical significance. Source data are provided as a Source Data file.

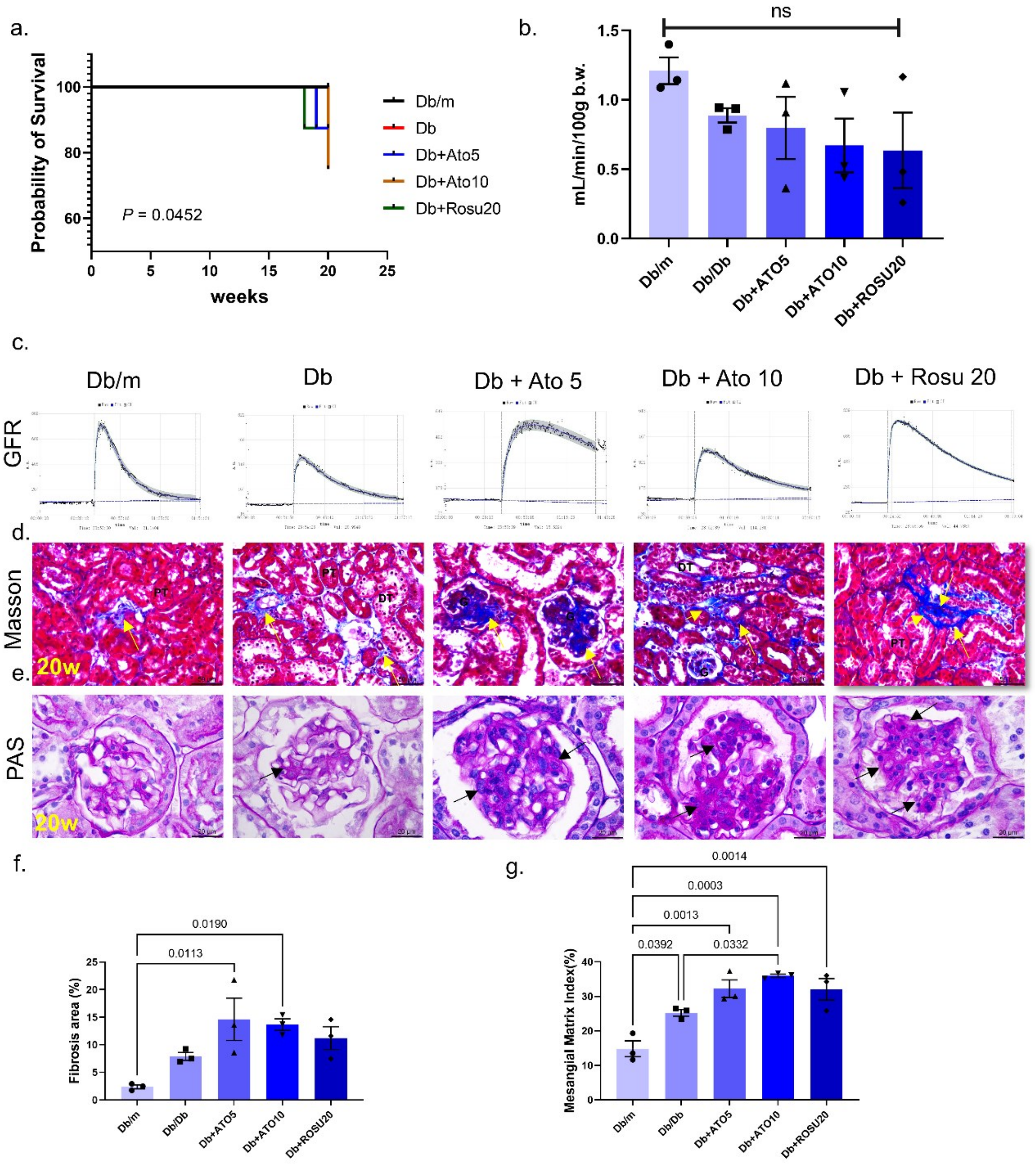

Supplementary Figure 4. Short-term statins administration similarly worsens renal injury in *db/db* mice. All mice were approximately 20 weeks

old. All mice were administered statins for 10 weeks. **(a)** Survival curve of *db/db* mice after short-term administration of statins. *n* = 8 in each group. **(b-c)** GFR measurement and representative images. *n* = 3 in each group. Data are expressed as means ± SEM (panel b-c). **(d, e)** Representative Masson’s trichrome staining and PAS staining. Masson’s trichrome staining original magnification ×400. Scale bar:50 μm. Yellow arrows indicate collagen deposition (blue). PAS staining original magnification ×1000. Scale bar: 20 μm. Black arrows indicate glomerular mesangial expansion. **(f, g)** Masson’s trichrome staining and PAS staining quantification. All image part of the kidney was cortex. Data are expressed as means ± SEM. *n* = 3 in each group. One-way ANOVA with Tukey post hoc test was used for the analysis of statistical significance. Source data are provided as a Source Data file.

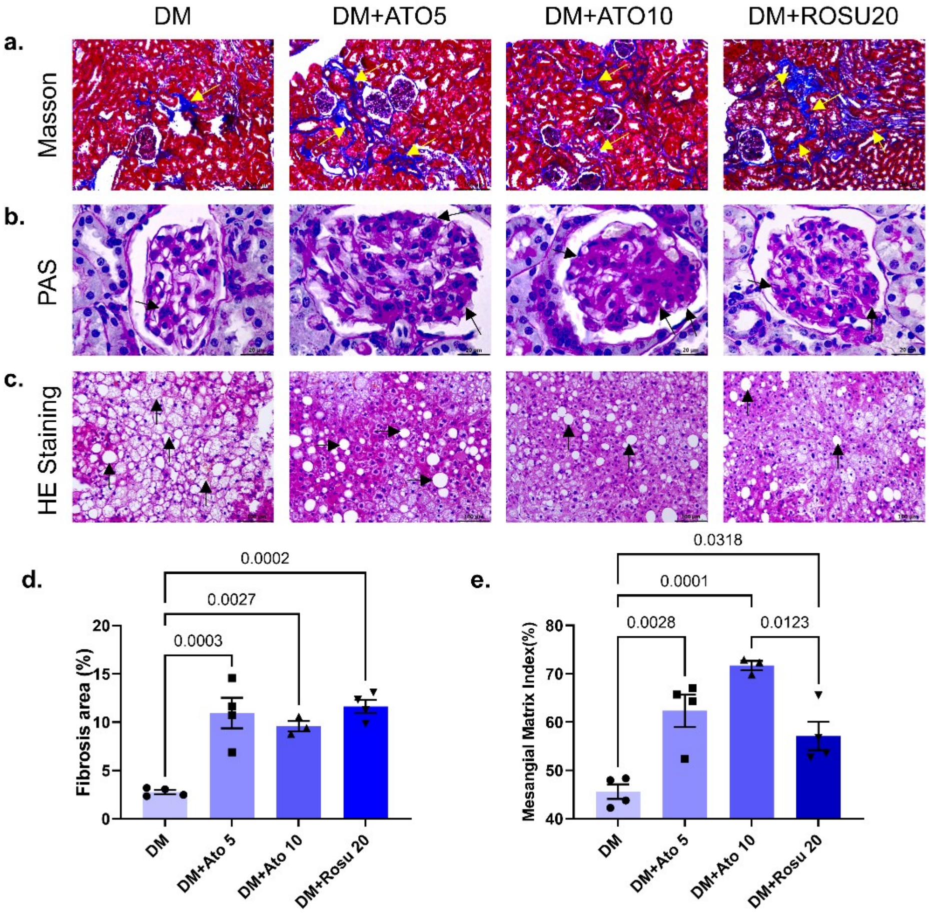

Supplementary Figure 5. Long-term statins administration worsens renal injury but ameliorated lipid accumulation in the liver of KK-ay diabetic mice. All mice were approximately 50 weeks old. **(a)** Representative sections of Masson's trichrome staining. Original magnification ×200. Scale bar:100 μm. **(b)** Representative sections of PAS staining for glomerulus. Arrows point to areas of glomerular mesangial expansion. Original magnification ×1000. Scale bar: 20 μm. **(c)** Representative images of HE staining for liver. Arrows represent large lipid droplets are present in hepatocytes. Original magnification ×200. Scale bar: 100μm. **(d)** Quantification of tubulointerstitial fibrosis in the kidney cortex. **(e)** Quantification of the mesangial area glomerulus. Data are expressed as means ± SEM. *n* = 4 in each group. One-way ANOVA with Tukey post hoc

test was used for the analysis of statistical significance. Source data are provided as a Source Data file.

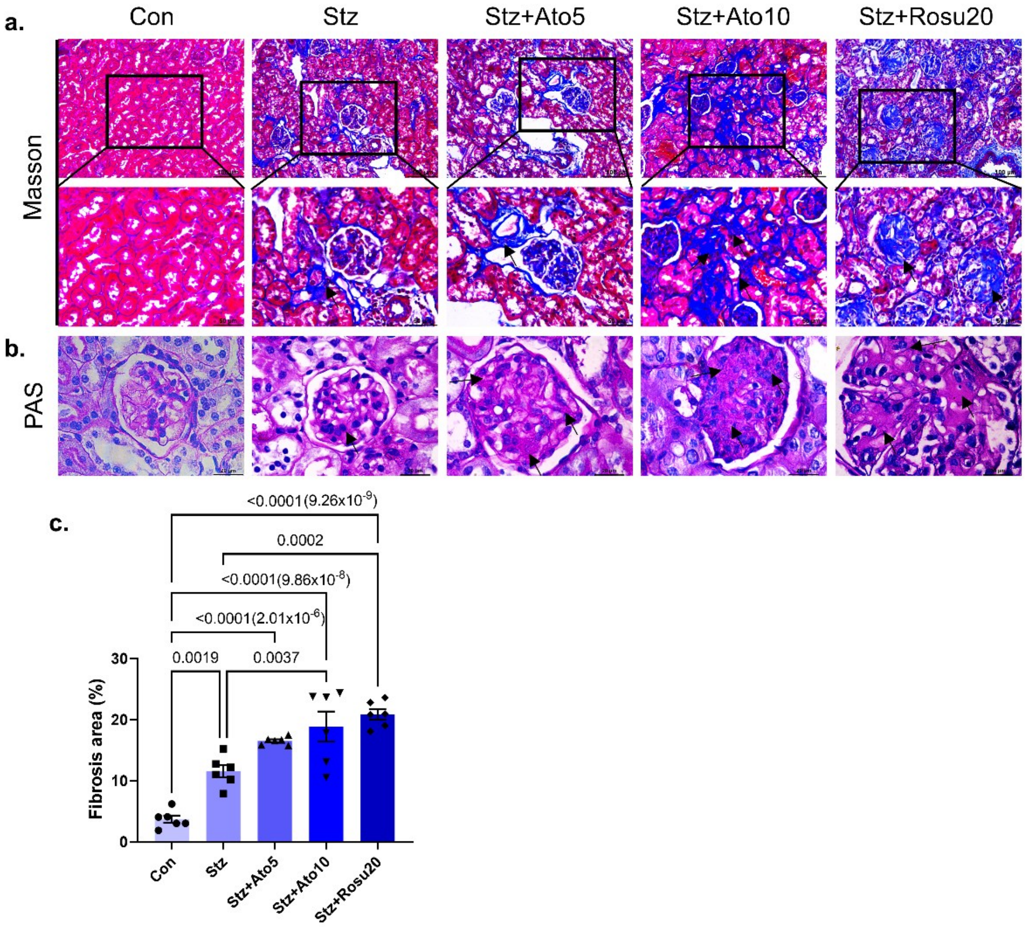

Supplementary Figure 6. Long-term statins administration worsens renal injury in STZ -induced diabetic mice. All mice were approximately 50 weeks old. **(a)** Representative sections of Masson’s trichrome staining. In the picture, collagenous components are stained as blue color and cytoplasm is varying shades of red. Collagen deposits (blue) are evident within the fibrotic interstitial lesions between tubular cells, and even in the glomerulus, shown as arrows. Original magnification ×200 or ×400. Scale bar: 100 μm or 50 μm. **(b)** Representative sections of PAS staining for glomerulus. Arrows point to areas

of glomerular mesangial expansion. Original magnification ×1000. Scale bar: 20 μm. **(c)** Quantification of tubulointerstitial fibrosis in the kidney cortex. All image part of the kidney was cortex. Data are expressed as means ± SEM. *n* = 6 in each group. One-way ANOVA with Tukey post hoc test was used for the analysis of statistical significance. Source data are provided as a Source Data file.

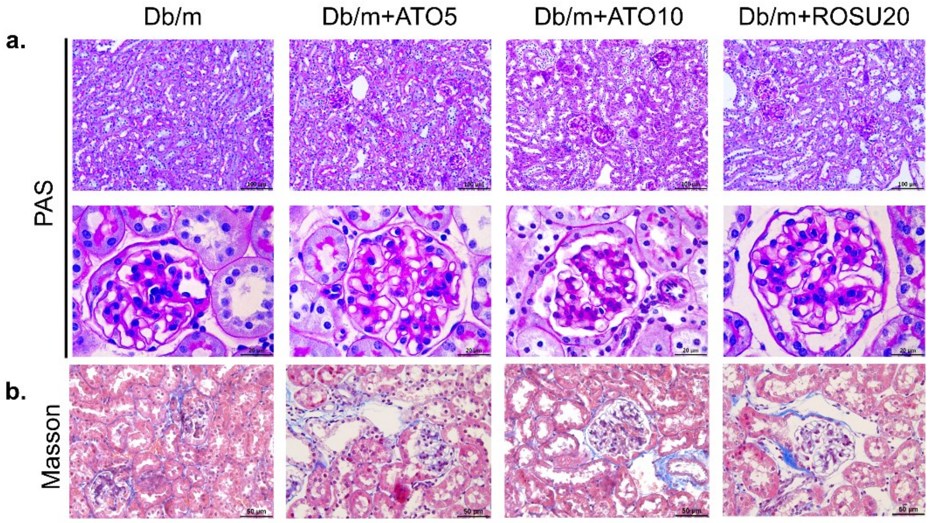

Supplementary Figure 7. Pathological characteristics of long-term statins administration in the kidney of *db/m* mice. All mice were approximately 50 weeks old. **(a)** Representative sections of PAS staining. PAS staining showed no significant pathological changes in *db/m* mice after long-term statins administration, such as glomerular hypertrophy, visible glomerular mesangial expansion. Original magnification ×200 and ×1000. Scale bar: 100 μm and 20 μm. **(b)** Representative sections of Masson's trichrome staining. Masson's trichrome staining showed that long-term statins administration did not aggravate renal fibrosis in *db/m* mice. Original magnification ×400. Scale bar:

50 μm. All image part of the kidney was cortex. *n* = 6 in each group. Source data are provided as a Source Data file.

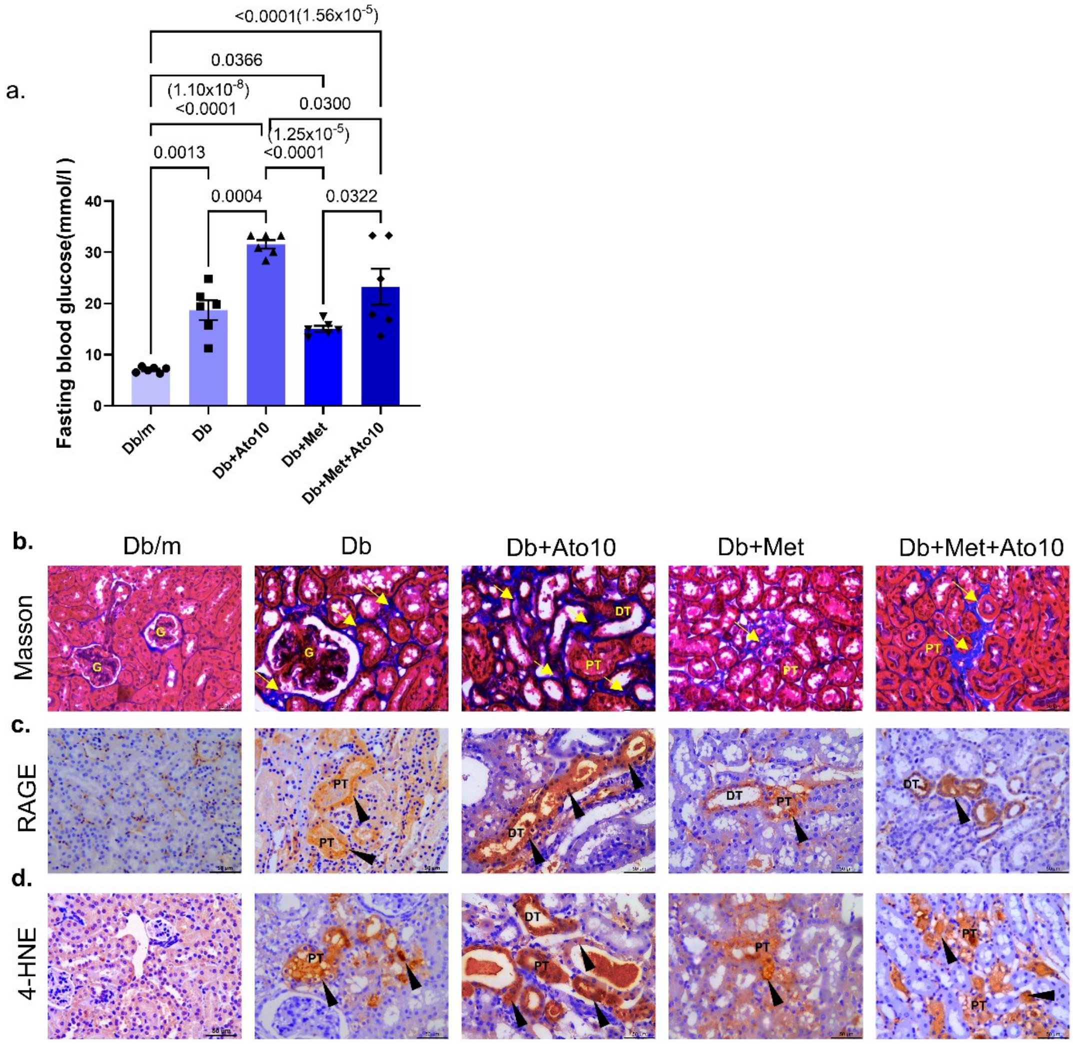

Supplementary Figure 8. Long-term statins administration combined with metformin alleviated renal injury in *db/db* mice. All mice were approximately 50 weeks old. **(a)** Measurements of fasting blood glucose. *n* = 6 in each group. **(b)** Representative sections of Masson's trichrome staining. In the picture, collagenous components are stained as blue color and cytoplasm is varying shades of red. Collagen deposits (blue) are evident within the fibrotic interstitial

lesions between tubular cells, and even in the glomerulus, shown as arrows. Original magnification ×400. Scale bar: 50 μm. **(c)** Immunohistochemical images of the RAGE in kidney sections. RAGE is mainly expressed on the cell membrane and cytoplasmic, and the specific location is indicated by the arrows. Original magnification ×400. Scale bar: 50 μm. **(d)** Immunohistochemical images of the 4-HNE in kidney sections. 4-HNE is mainly expressed in the cytoplasmic, and the specific location is indicated by the arrows. Original magnification ×400. Scale bar: 50 μm. All image part of the kidney was cortex. Renal structures indicated as glomerulus (G), proximal tubule (PT), and distal tubule (DT). Data are expressed as means ± SEM. One-way ANOVA with Tukey post hoc test was used for the analysis of statistical significance. Source data are provided as a Source Data file.

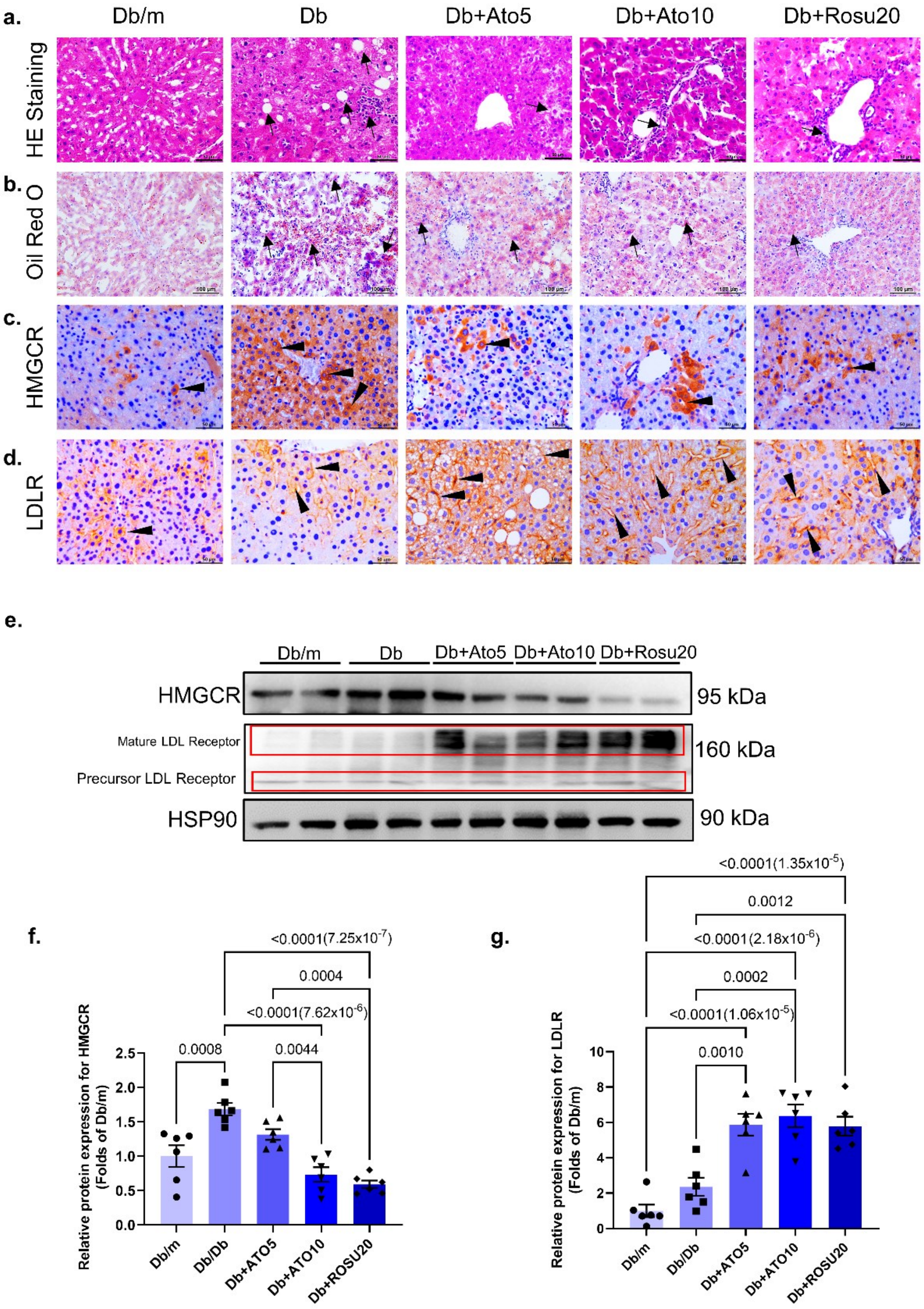

Supplementary Figure 9. Long-term statins administration ameliorated lipid accumulation in the liver of *db/db* mice. All mice were approximately 50 weeks old. **(a)** Representative image of HE staining. Arrows point to areas of hepatocyte steatosis and inflammatory cell infiltration. Original magnification

×400. Scale bar: 50 μm. **(b)** Representative images of Oil Red O staining. Lipid droplets appear as red spots, show as arrows. Original magnification ×200. Scale bar: 100 μm. **(c)** Immunohistochemistry of HMGCR staining. HMGCR is mainly expressed on the cytoplasmic of hepatocyte, and the specific location is indicated by the arrows. Original magnification ×400. Scale bar: 50 μm. **(d)** Immunohistochemistry of LDLR staining. LDLR mainly expressed on the cell membrane of hepatocyte, and the specific location is indicated by the arrows. Original magnification ×400. Scale bar: 50 μm. **(e)** the immunoblot analysis of HMGCR and LDLR. **(f-g)** Analysis of the grayscale image between them. Data are expressed as means ± SEM. *n* = 6 in each group. One-way ANOVA with Tukey post hoc test was used for the analysis of statistical significance. Source data are provided as a Source Data file.

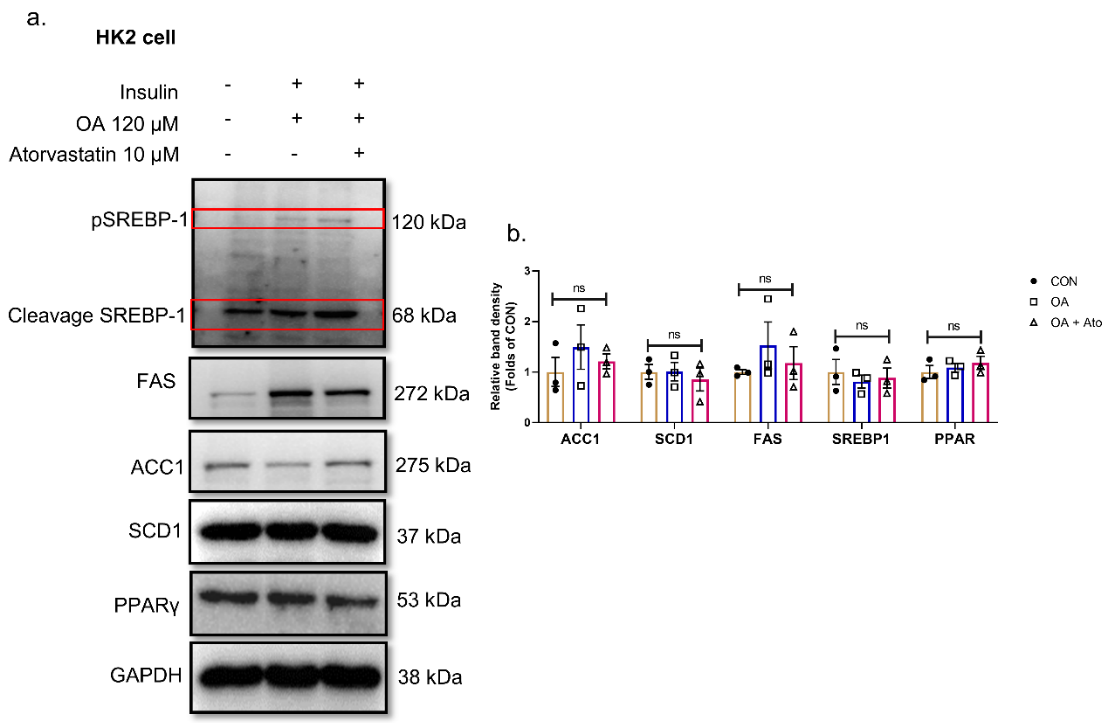

Supplementary Figure 10. Statins cannot increase the SREBP-1 in HK-2 cells. The HK-2 cell treated with OA 120 μM and insulin 2.4 μM, treated with or without statin 10 mM for 24 hours. **(a)** The immunoblot analysis of SREBP-1, FAS, SCD1, ACC1, PPARγ in HK-2 cells. **(b)** Analysis of the grayscale image between them. Representative blots from three biologically independent samples/group were combined from three independent experiments. Data are expressed as means ± SEM. Data are expressed as means ± SEM. One-way ANOVA with Tukey post hoc test was used for the analysis of statistical significance. Source data are provided as a Source Data file.
