# Supplementary material for: Long-term statins administration exacerbates diabetic nephropathy via ectopic fat deposition in diabetic mice
Source: Nat Commun. 2023 Jan 24;14:390. doi: 10.1038/s41467-023-35944-z (PMC9873739; doi:10.1038/s41467-023-35944-z)
Supplement: Supplementary file 3 — Reporting Summary [file 41467_2023_35944_MOESM3_ESM.pdf]

nature portfolio

Corresponding author(s): Weibin Cai
Last updated by author(s): Dec 12, 2022

Reporting Summary

Nature Portfolio wishes to improve the reproducibility of the work that we publish. This form provides structure for consistency and transparency in reporting. For further information on Nature Portfolio policies, see our Editorial Policies and the Editorial Policy Checklist.

Statistics

For all statistical analyses, confirm that the following items are present in the figure legend, table legend, main text, or Methods section.

n/a	Confirmed	
☐	☒	The exact sample size (*n*) for each experimental group/condition, given as a discrete number and unit of measurement
☐	☒	A statement on whether measurements were taken from distinct samples or whether the same sample was measured repeatedly
☐	☒	The statistical test(s) used AND whether they are one- or two-sided<br>*Only common tests should be described solely by name; describe more complex techniques in the Methods section.*
☒	☐	A description of all covariates tested
☐	☒	A description of any assumptions or corrections, such as tests of normality and adjustment for multiple comparisons
☐	☒	A full description of the statistical parameters including central tendency (e.g. means) or other basic estimates (e.g. regression coefficient) AND variation (e.g. standard deviation) or associated estimates of uncertainty (e.g. confidence intervals)
☐	☒	For null hypothesis testing, the test statistic (e.g. *F*, *t*, *r*) with confidence intervals, effect sizes, degrees of freedom and *P* value noted<br>*Give P values as exact values whenever suitable.*
☒	☐	For Bayesian analysis, information on the choice of priors and Markov chain Monte Carlo settings
☒	☐	For hierarchical and complex designs, identification of the appropriate level for tests and full reporting of outcomes
☒	☐	Estimates of effect sizes (e.g. Cohen's *d*, Pearson's *r*), indicating how they were calculated

Our web collection on statistics for biologists contains articles on many of the points above.

Software and code

Policy information about availability of computer code

Data collection	GFR was performed in a NIC-Kidney device (MediBeacon, Germany). FFAs uptake was measured by a small animal living fluorescence imaging system (Xtrem, Bruker, Germany). Immunohistochemistry of tissues was examined and photographed with a microscope (DFC700T, Leica, Germany). Immunofluorescence images of tissues, cells images were obtained using a confocal microscopy (LSM 800, Zeiss). Transmission electron microscopy (TEM) was examined and photographed with a transmission electron microscope (Tecnai G2 Spirit Twin +GATAN 832.10W; FEI; Czech Republic). Electrophoretic techniques were performed in a PowerPac/Mini (BIO-RAD, California, USA), and exposure of immunoblotting was performed in a BIO-RAD imaging system (BIO-RAD, California, USA). The RNA library was sequenced on the BGISEQ-500 platform (BGI Genomics, Shenzhen, China). Optical density values were collected on a mutilskan go microplate reader (Thermo Fisher Scientific, Massachusetts, USA).
Data analysis	Quantifications of immunoblotting, immunofluorescence staining, and immunohistochemistry staining were analyzed by Image J (version 1.45) (National Institutes of Health, Bethesda, MD, USA) and Image-Pro Plus version 6.0 software□Media Cybernetics, Inc. , Rockville, MD, USA). Statistical analysis was performed with the SPSS Statistics v.22.0 (IBM Corp.), GraphPad Prism 9.2 (Inc., La Jolla, CA) and R-4.2.2. Virtual screening was performed using the Surflex-Dock of SYBYL-X 2.0 (Tripos). The Biorender.com was used for produce Fig 10. Adobe Photoshop CS6 software (California, USA) was used for image layout.

For manuscripts utilizing custom algorithms or software that are central to the research but not yet described in published literature, software must be made available to editors and reviewers. We strongly encourage code deposition in a community repository (e.g. GitHub). See the Nature Portfolio guidelines for submitting code & software for further information.

Data

Policy information about availability of data

All manuscripts must include a data availability statement. This statement should provide the following information, where applicable:

- Accession codes, unique identifiers, or web links for publicly available datasets
- A description of any restrictions on data availability
- For clinical datasets or third party data, please ensure that the statement adheres to our policy

Source data contained the raw data underlying the following types of display items: any reported means/averages in bar charts, and tables, and uncropped versions of any gels or blots, labelled with the relevant panel and identifying information. The affymetrix raw files generated in this study have been deposited in the GEO database under accession code GSE196701[https://www.ncbi.nlm.nih.gov/geo/query/acc.cgi?acc=GSE196701. Source data are provided with this paper.

Human research participants

Policy information about studies involving human research participants and Sex and Gender in Research.

Reporting on sex and gender	This study does not design human research.
Population characteristics	This study does not design human research.
Recruitment	This study does not design human research.
Ethics oversight	This study does not design human research.

Note that full information on the approval of the study protocol must also be provided in the manuscript.

Field-specific reporting

Please select the one below that is the best fit for your research. If you are not sure, read the appropriate sections before making your selection.

☒ Life sciences ☐ Behavioural & social sciences ☐ Ecological, evolutionary & environmental sciences

For a reference copy of the document with all sections, see nature.com/documents/nr-reporting-summary-flat.pdf

Life sciences study design

All studies must disclose on these points even when the disclosure is negative.

Sample size	Because the individual differences in diabetic mice are large, if the sample size is not enough, it is easy to have large differences within the group. Therefore, we based on pre-test and previous publish experiments [Int J Mol Med. 2011 Nov;28(5):683-7.] to select sample sizes, which leading to determine statistically significant effects.
Data exclusions	No data were excluded.
Replication	Most expriments were replicated at least two times. All replications confirmed the findings.
Randomization	All mice enrolled in our study were of the same week of age and of similar body weight, and then randomly assigned to specific treatment groups.
Blinding	PAS staining, immunofluorescence staining, and immunohistochemistry staining and quantification was double-blinded in collect images and quantification using ImageJ software or Image-Pro Plus version 6.0 software. Other data analysis was performed in a simultaneous and unbiased manner for all collected samples, where no blinding was necessary.

Reporting for specific materials, systems and methods

We require information from authors about some types of materials, experimental systems and methods used in many studies. Here, indicate whether each material, system or method listed is relevant to your study. If you are not sure if a list item applies to your research, read the appropriate section before selecting a response.

Materials & experimental systems

n/a	Involved in the study
☐	☒ Antibodies
☐	☒ Eukaryotic cell lines
☒	☐ Palaeontology and archaeology
☐	☒ Animals and other organisms
☒	☐ Clinical data
☒	☐ Dual use research of concern

Methods

n/a	Involved in the study
☒	☐ ChIP-seq
☒	☐ Flow cytometry
☒	☐ MRI-based neuroimaging

Antibodies

Antibodies used

α-SMA (Cell signaling technology, 19245S, 1:200 for IHC), COL1A1 (Cell signaling technology, 72026S, 1:200 for IHC), NF-κB (Cell signaling technology, 8242S, 1:150 for IF), CD68 (Cell signaling technology, 97778S, 1:100 for IHC), 4-HNE (Abcam, ab46545, 1:100 for IHC), IL-1β (Cell signaling technology, 12242S, 1:1000 for WB, 1:100 for IHC), NGAL (Abcam, ab125075, 1:1000 for WB, 1:100 for IHC), SREBP-1 (Novus Biologicals, NB100-2215, 1:1000 for WB, 1:200 for IHC), Akt (pan) (C67E7) (Cell signaling technology, 4691S, 1:1000 for WB)?Phospho-Akt (Ser473) (Cell signaling technology, 4060S, 1:1000 for WB), Phospho-Akt (Thr308) (244F9) (Cell signaling technology, 4056S, 1:1000 for WB), FAS (Cell signaling technology, 3180S, 1:1000 for WB, 1:100 for IHC), SCD1 (Cell signaling technology, 2794S, 1:1000 for WB, 1:100 for IHC), ACC1 (Proteintech, 21923-1-AP, 1:1000 for WB), Phospho-p70 S6 Kinase (Thr389) (Cell signaling technology, 9205S, 1:1000 for WB), p70(S6K) Polyclonal antibody (Proteintech, 14485-1-AP, 1:1000 for WB), PI3 Kinase p85 (19H8) (Cell signaling technology, 4257S, 1:1000 for WB), Phospho-PI3 Kinase p85 (Tyr458)/p55 (Tyr199) (Cell signaling technology, 4228S, 1:1000 for WB), ATGL (30A4) (Cell signaling technology, 2439S, 1:1000 for WB), CD36(Sigma Aldrich, HPA002018, 1:1000 for WB, 1:100 for IHC), anti-HMGCR(Abcam, ab242315, 1:1000 for WB, 1:100 for IHC), CPT1A(Cell signaling technology, 12252S, 1:1000 for WB), RAGE(Proteintech, 16346-1-AP, 1:100 for IHC), LDLR (Proteintech, 10785-1-AP, 1:1000 for WB, 1:100 for IHC), Anti-Wilms Tumor (Abcam, ab89901, 1:100 for IF), Anti-Nephrin (R&D systems, AF3159, 1:100 for IF), PPARγ (81B8) (Cell signaling technology, 2443S, 1:1000 for WB), BODIPY™ 493/503 (Thermo fisher scientific, D3922, 1:1000 for IF); GAPDH(Proteintech, 60004-1-Ig, 1:1000 for WB); HSP90 (Proteintech, 13171-1-AP, 1:1000 for WB); HRP-conjugated Affinipure Goat Anti-Rabbit IgG(H+L) (Proteintech, SA00001-2, 1:2000 for WB); HRP-conjugated Affinipure Goat Anti-Mouse IgG(H+L) (Proteintech, SA00001-1, 1:2000 for WB); Donkey anti-Mouse IgG (H+L) Highly Cross-Adsorbed Secondary Antibody, Alexa Fluor™ 594 (Invitrogen, A-21203, 1:2000 for IF); Donkey anti-Rabbit IgG (H+L) Highly Cross-Adsorbed Secondary Antibody, Alexa Fluor™ 594 (Invitrogen, A-21207, 1:2000 for IF); Donkey anti-Rabbit IgG (H+L) Highly Cross-Adsorbed Secondary Antibody, Alexa Fluor™ 488 (Invitrogen, A-21206, 1:2000 for IF).

Validation

Antibody validation information can be found on manufacturers' website:
α-Smooth Muscle Actin (D4K9N) XP® Rabbit mAb #19245: https://www.cellsignal.cn/products/primary-antibodies/a-smooth-muscle-actin-d4k9n-xp-rabbit-mab/19245;
COL1A1 (E8F4L) XP® Rabbit mAb #72026: https://www.cellsignal.cn/products/primary-antibodies/col1a1-e8f4l-xp-rabbit-mab/72026;
NF-κB p65 (D14E12) XP® Rabbit mAb #8242: https://www.cellsignal.cn/products/primary-antibodies/nf-kb-p65-d14e12-xp-rabbit-mab/8242;
CD68 (E3O7V) Rabbit mAb #97778: https://www.cellsignal.cn/products/primary-antibodies/cd68-e3o7v-rabbit-mab/97778;
Anti-4 Hydroxynonenal Rabbit mAb: https://www.abcam.cn/4-hydroxynonenal-antibody-ab46545.html;
IL-1β (3A6) Mouse mAb #12242: https://www.cellsignal.cn/products/primary-antibodies/il-1b-3a6-mouse-mab/12242;
Anti-Lipocalin-2 / NGAL Rabbit mAb: https://www.abcam.cn/lipocalin-2--ngal-antibody-epr5084-ab125075.html;
SREBP1 Rabbit Antibody: https://www.novusbio.com/products/srebp1-antibody_nb100-2215;
Akt (pan) (C67E7) Rabbit mAb #4691: https://www.cellsignal.cn/products/primary-antibodies/akt-pan-c67e7-rabbit-mab/4691;
Phospho-Akt (Ser473) (D9E) XP® Rabbit mAb #4060: https://www.cellsignal.cn/products/primary-antibodies/phospho-akt-ser473-d9e-xp-rabbit-mab/4060;
Phospho-Akt (Thr308) (244F9) Rabbit mAb #4056: https://www.cellsignal.cn/products/primary-antibodies/phospho-akt-thr308-244f9-rabbit-mab/4056;
Fatty Acid Synthase (C20G5) Rabbit mAb #3180: https://www.cellsignal.cn/products/primary-antibodies/fatty-acid-synthase-c20g5-rabbit-mab/3180;
SCD1 (C12H5) Rabbit mAb #2794: https://www.cellsignal.cn/products/primary-antibodies/scd1-c12h5-rabbit-mab/2794;
ACC1 Polyclonal Rabbit antibody: https://www.ptgcn.com/products/ACACA-Antibody-21923-1-AP.htm;
Phospho-p70 S6 Kinase (Thr389) Antibody #9205: https://www.cellsignal.cn/products/primary-antibodies/phospho-p70-s6-kinase-thr389-antibody/9205;
p70(S6K) Polyclonal Rabbit antibody: https://www.ptgcn.com/products/p70(S6K)-Antibody-14485-1-AP.htm;
PI3 Kinase p85 (19H8) Rabbit mAb #4257: https://www.cellsignal.cn/products/primary-antibodies/pi3-kinase-p85-19h8-rabbit-mab/4257;
Phospho-PI3 Kinase p85 (Tyr458)/p55 (Tyr199) Antibody #4228: https://www.cellsignal.cn/products/primary-antibodies/phospho-pi3-kinase-p85-tyr458-p55-tyr199-antibody/4228;
ATGL (30A4) Rabbit mAb #2439: https://www.cellsignal.cn/products/primary-antibodies/atgl-30a4-rabbit-mab/2439;
Anti-CD36 antibody produced in rabbit: https://www.sigmaaldrich.cn/CN/zh/product/sigma/hpa002018;
Anti-HMGCR antibody [CL0260] (ab242315): https://www.abcam.cn/hmgcr-antibody-cl0260-ab242315.html;
CPT1A (D3B3) Rabbit mAb #12252: https://www.cellsignal.cn/products/primary-antibodies/cpt1a-d3b3-rabbit-mab/12252;
AGER(RAGE) Polyclonal Rabbit antibody: https://www.ptgcn.com/products/AGER-Antibody-16346-1-AP.htm;
LDLR Polyclonal Rabbit antibody: https://www.ptgcn.com/products/LDLR-Antibody-10785-1-AP.htm;
Anti-Wilms Tumor Rabbit antibody [CAN-R9(IHC)-56-2] (ab89901): https://www.abcam.cn/wilms-tumor-protein-antibody-can-r9ihc-56-2-ab89901.html;
Mouse Nephrin Antibody: https://www.rndsystems.com/cn/products/mouse-nephrin-antibody_af3159;
PPARγ (81B8) Rabbit mAb #2443: https://www.cellsignal.cn/products/primary-antibodies/pparg-81b8-rabbit-mab/2443;
BODIPY™ 493/503: https://www.thermofisher.cn/order/catalog/product/D3922?SID=srch-srp-D3922

GAPDH Monoclonal antibody: https://www.ptgcn.com/products/GAPDH-Antibody-60004-1-Ig.htm;
HSP90 Polyclonal antibody: https://www.ptgcn.com/products/HSP90-Antibody-13171-1-AP.htm;
HRP-conjugated Affinipure Goat Anti-Rabbit IgG(H+L): https://www.ptgcn.com/products/HRP-conjugated-Affinipure-Goat-Anti-Rabbit-IgG-H-L-secondary-antibody.htm;
HRP-conjugated Affinipure Goat Anti-Mouse IgG(H+L): https://www.ptgcn.com/products/HRP-conjugated-Affinipure-Goat-Anti-Mouse-IgG-H-L-secondary-antibody.htm;
Donkey anti-Mouse IgG (H+L) Highly Cross-Adsorbed Secondary Antibody, Alexa Fluor™ 594: https://www.thermofisher.cn/cn/zh/antibody/product/Donkey-anti-Mouse-IgG-H-L-Highly-Cross-Adsorbed-Secondary-Antibody-Polyclonal/A-21203;
Donkey anti-Rabbit IgG (H+L) Highly Cross-Adsorbed Secondary Antibody, Alexa Fluor™ 594: https://www.thermofisher.cn/cn/zh/antibody/product/Donkey-anti-Rabbit-IgG-H-L-Highly-Cross-Adsorbed-Secondary-Antibody-Polyclonal/A-21207;
Donkey anti-Rabbit IgG (H+L) Highly Cross-Adsorbed Secondary Antibody, Alexa Fluor™ 488: https://www.thermofisher.cn/cn/zh/antibody/product/Donkey-anti-Rabbit-IgG-H-L-Highly-Cross-Adsorbed-Secondary-Antibody-Polyclonal/A-21206.

Eukaryotic cell lines

Policy information about cell lines and Sex and Gender in Research

Cell line source(s)	Human proximal tubular cells (HK2 cells) (presented by Prof. Weidong Wang, Zhongshan School of Medicine, Sun Yat-sen University) were obtained from ATCC.
Authentication	HK2 cells line was authenticated using Short Tandem Repeat (STR) analysis.
Mycoplasma contamination	The cell-lines used were routinely tested negative for mycoplasma.
Commonly misidentified lines (See ICLAC register)	Not used.

Animals and other research organisms

Policy information about studies involving animals; ARRIVE guidelines recommended for reporting animal research, and Sex and Gender in Research

Laboratory animals	Db/m (C57BLKS/J background), db/db mice(C57BLKS/J background) and srebp1-deficient mice(C57BL/6JGpt background)(Strain NO. T037279) were purchased from GemPharmatech Co., Ltd (Jiangsu, China) , KK-Ay mice were obtained from Beijing HFK Bioscience Co. Ltd. (Beijing, China). ApoE−/− mice (C57BL/6JGpt background) and LDLR -/- mice (C57BL/6JGpt background) were obtained from Jackson Laboratory.C57BL/6J mice were purchased from Laboratory Animal Center of Sun Yat-sen University. All enrolled mice were male and aged 8-10 weeks. Mice maintained at the Center for Disease Model Animals of Sun Yat-sen University. Mice were housed on a 12 h light-dark cycle at 22–25 °C with 40–70% humidity and allowed free access to food and water except as noted.
Wild animals	The study did not involve wild animals.
Reporting on sex	In general, the reason for using male mice in animal experiments is to avoid interference with the female estrous cycle, but for our study, estrogen in female mice has an impact on studying diabetes. Therefore, only male mice were used in our animal studies.
Field-collected samples	The study did not involve field-collected animals.
Ethics oversight	All animal experiments were performed according to the regulations approved by the Animal Care and Ethics Committee of Sun Yat-sen University (NO.2021000957).

Note that full information on the approval of the study protocol must also be provided in the manuscript.
